# Dasatinib overrides the differentiation blockage in a patient with mutant-*KIT* D816V positive CBFβ-MYH11 leukemia

**DOI:** 10.18632/oncotarget.24376

**Published:** 2018-01-31

**Authors:** Kerstin M. Kampa-Schittenhelm, Wichard Vogel, Irina Bonzheim, Falko Fend, Marius Horger, Lothar Kanz, Martin Soekler, Marcus M. Schittenhelm

**Affiliations:** ^1^ Department of Oncology, Hematology, Rheumatology, Clinical Immunology and Pulmonology, University Hospital Tübingen, Tübingen, Germany; ^2^ Institute of Pathology and Neuropathology, Reference Center for Hematopathology, University Hospital Tübingen, Tübingen, Germany; ^3^ Department of Diagnostic and Interventional Radiology, University Hospital Tübingen, Tübingen, Germany

**Keywords:** CBF AML, KIT, tyrosine kinase inhibition, differentiation

## Abstract

Activating *KIT* D816V mutations are frequently found in CBF AML, which predicts for an unfavorable outcome. Dasatinib is a potent inhibitor of wildtype and mutant-KIT isoforms – including D816V. We now provide proof of antileukemic efficacy in a patient with relapsing mutant-*KIT* D816V CBF AML. Importantly, this effect is mediated via overriding the differentiation blockage of the leukemia clone.

In addition, we show that dasatinib is capable to induce pulmonary differentiation syndrome – and therefore needs close monitoring of patients under therapy.

## INTRODUCTION

Activating *KIT* D816V mutations are frequently found in core binding factor (CBF) AML [1], predicting for an unfavorable outcome [2]. Furthermore, it has been shown that *KIT* D816V functions as a second step driver mutation contributing to leukemogenesis in CBF subentities [3].

In analogy to mutant-FLT3 isoforms in AML, mutant-KIT D816 isoforms may provide an attractive therapeutic target in CBF AML. Mutant FLT3 is extensively studied using tyrosine kinase inhibitors (TKI) – and the first TKI, midostaurin, just recently gained FDA approval in this setting. However, there are no approved targeted therapeutics for mutant-KIT CBF AML so far.

We have previously shown that the BCR-ABL1 tyrosine kinase inhibitor dasatinib is a potent inhibitor of wildtype- and mutant-KIT, including the KIT D816V isoform, resulting in potent antiproliferative and proapoptotic efficacy [4]. Dasatinib is currently being tested in combination with a chemotherapy backbone in clinical trials for the treatment of core binding factor leukemias (NCT02013648, NCT00850382, NCT02113319).

We now provide proof of antileukemic efficacy in a patient with relapsed mutant-*KIT* D816V positive CBF AML – and point to an unexpected mode of action via release of the differentiation blockage and maturation of leukemia blasts.

## RESULTS AND DISCUSSION

A 77-year-old unfit male patient with CBFβ-MYH11 positive AML (46,XY,inv(16)(q13q22) [25], *KIT* p.Asp816Val (exon 17)) relapsed after effective initial treatment with decitabine (hematologic CR after 4 cycles, total of 12 cycles administered) and presented with beginning leukocytosis. Due to the very limited therapeutic options in this frail patient and a high *KIT* D816V mutation burden at relapse (75,8% *KIT*^mut^/*KIT*^WT^ and 73% *CBFβ-MYH11/ABL1* compared to 3,8% *KIT*^mut^/*KIT*^WT^ and 4,07% *CBFβ-MYH11/ABL1* in a bone marrow aspirate at hematologic CR while under decitabine - indicating outgrowth of the mutant-*KIT* clone at relapse) an individual attempt with the KIT inhibitor dasatinib (Sprycel^®^) was initiated.

**Figure 1:**
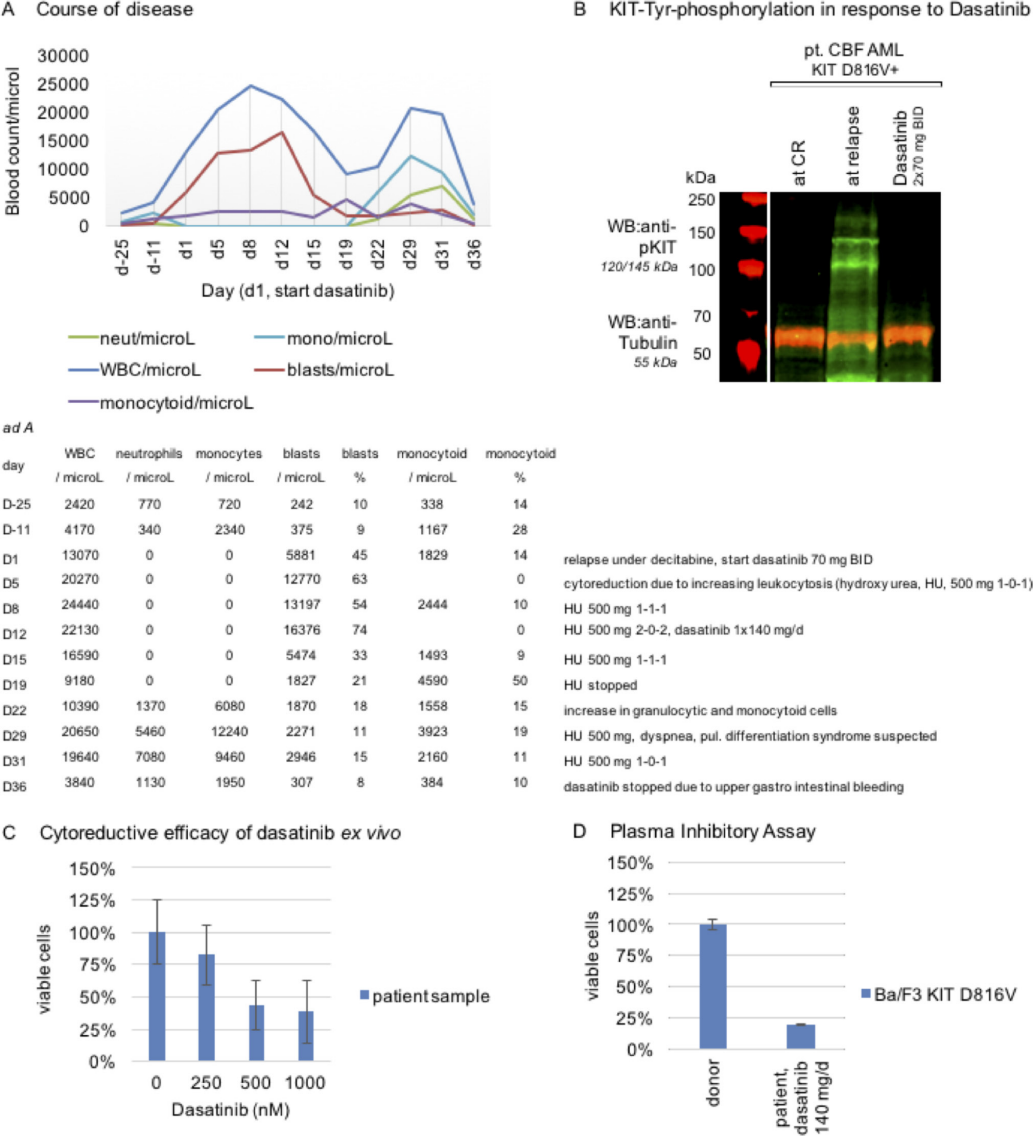
Target specificity of dasatinib and course of disease in a patient with mutant-KIT CBF AML (**A**) Course of disease: 77-year-old unfit male patient with relapsing disease and high *KIT* D816V mutation burden. *Ad A* absolute cell counts. (**B**) Proof of on-target efficacy of dasatinib in patient mononuclear cell samples, isolated by Ficoll Hypaque density gradient fractionation, at CR (while under decitabine therapy), at relapse and while under stable dasatinib therapy: Western immunoblotting shows potent inhibition of KIT tyrosine-phosphorylation (Tyr917) upon dasatinib exposure *in vivo*. Tubulin serves as loading control. (Assessment on a LI-COR Odyssey^®^ fluorescence optical system). (**C**) Mimicking cytoreductive capacity of dasatinib *ex vivo*: A patient sample is treated with dasatinib in dose dilution assays and reduction of the viable cohort after 48 hours is assessed flow cytometrically and depicted in a normalized dose-effect bar graph (technical quintuplicate). (**D**) Plasma inhibitory assay: patient serum, while under stable therapy with dasatinib 140 mg/d, is used to culture Ba/F3 *KIT* D816V cells and reduction of the viable cohort is assessed flow cytometrically and depicted in a normalized dose-effect bar graph (technical triplicate). Serum of a healthy drug-naive donor is used as a negative control.

Therapy was started with dasatinib 70 mg BID after informed consent (Figure 1A). Target-specificity of dasatinib was confirmed in a Western immunoblot using patient’s samples at complete remission while under therapy with decitabine (absence of phospho-KIT in the mononuclear cell fraction), at relapse (strong KIT phosphorylation), and while under treatment with dasatinib 2 × 70 mg/day (potent inhibition of KIT tyrosine phosphorylation, i.e. inactivation of KIT, Figure 1B).

In addition, we tested the cytoreductive capacity of dasatinib in a sample of this patient at relapse after decitabine treatment – and demonstrate *ex vivo* efficacy of dasatinib. A dose-dilution bar graph is provided with Figure 1C.

Nevertheless, and despite proven target-specificity of dasatinib, we did not observe prompt cytoreduction upon dasatinib, which may reflect high dynamics of the disease with evolving leukocytosis in this patient.

Consequently, on day 5, additional cytoreductive therapy with hydroxyurea (HU) was started (500 mg BID). As leukocytosis progressed, the dose of HU was increased on day 8 (500 mg TID) and day 12 (500 mg 2-0-2) accordingly. Additionally, dosing of dasatinib was changed to 140 mg once a day, in order to achieve higher serum peak levels, as shown previously [5].

The following days, a continuous decline of leukocyte counts was noted – and HU was reduced (500 mg TID day 15) and stopped (day 19). Dosing of dasatinib was not modified.

To cross-check, a plasma inhibitory assay (PIA), as an indirect indicator for clinically active doses of dasatinib achievable *in vivo,* was set up: Serum of the presented patient was collected while on steady therapy with dasatinib 140 mg/day (day 22) and Ba/F3 *KIT* D816V cells – a well-established reference cell line harboring the autoactivating *KIT* D816V mutation [4, 6, 7] – were cultured for 48 hours in the patient’s serum, as well as in serum of a healthy drug-naïve donor as a negative control. Cytoreduction was only observed in the patient’s serum compared to an unchanged viable mononuclear cell fraction in the donor control, arguing for clinically effective concentrations of dasatinib *in vivo* (Figure 1D).

**Figure 2:**
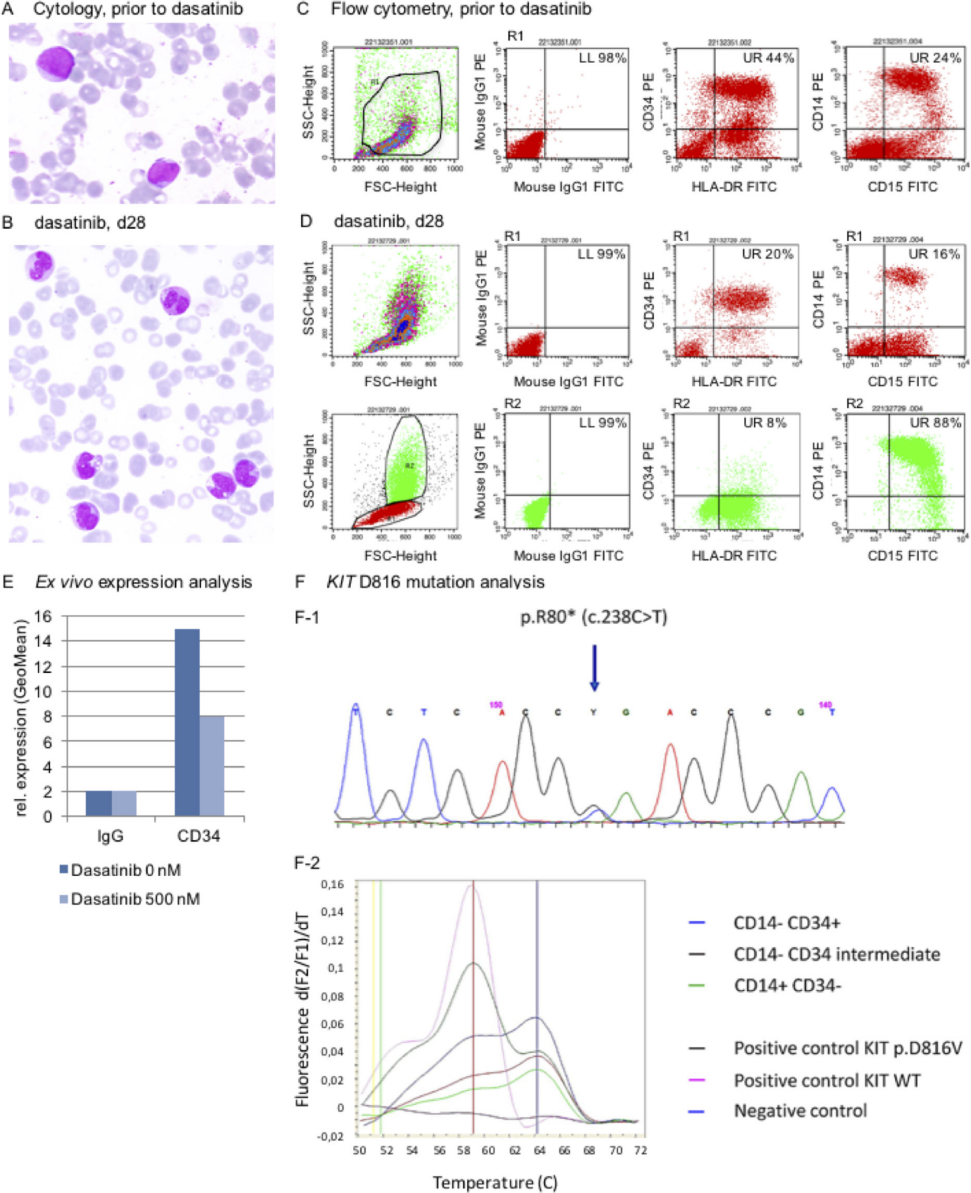
Release of the maturation blockage in leukemia cells in response to dasatinib (**A**, **B**) Cytology (confocal Zeiss^®^ AXIO Imager.A1 microscope): A May-Gruenwald-Giemsa stain shows signs of dysplastic maturation of granulo and monocytoid-like cells (A, untreated patient blood smear prior to start of treatment with dasatinib; B, 28-day on-dasatinib patient sample; zoom-in figures are provided as Supplementary Figure 1A, 1B). (**C**, **D**) Immunophenotyping argues for myelomonocytic maturation of the leukemia clone, shifting from a CD34+ positive phenotype (C) to CD34- negativity (D, R1 of upper quadrant plots (in red) refer to R1 in the FSC/SCC region plot (in red). R2 in the lower quadrant plots (in green) refer to R2 in the region plot (in green)), (Assessment on a FACScalibur^®^ flow cytometer using CellQuest^®^ software). (**E**) Release of differentiation block in response to dasatinib is mimicked in an *ex vivo* immunophenotyping assay demonstrating decrease of CD34 expression in a patient sample treated with dasatinib *ex vivo*. (**F**) A cell sort of the monocytoid CD14–/CD34+ vs. CD14–/CD34(+) vs. CD14+/CD34– populations was performed to screen for the *KIT* D816V mutation – which was detected in all cohorts (F-1, sanger sequencing; F-2 HPLC melting curve).

Intriguingly, starting on day 12, a steady decline of morphologic blasts in the peripheral blood was observed–going along with an emerging dysplastic granulocyte and monocyte population (Figure 2A, 2B, day 28). This observation led us to speculate that dasatinib resulted in release of the differentiation blockage of the leukemic clone *in vivo*.

Immunophenotyping underlined this notion, demonstrating a shift from a CD14+/CD15+/CD34+ positive phenotype to CD34 negativity (Figure 2C, 2D).

We therefore mimicked release of the differentiation blockage *ex vivo:* Native blasts (sample taken prior to start of dasatinib) were cultured and treated with dasatinib at IC50 concentrations and indeed, relative decrease of CD34 positivity was noted flow cytometrically 72 hours after drug exposure – again arguing for maturation of the patient’s blasts (Figure 2E).

To ultimately verify maturation of the original leukemia clone, a cell sort was set up using a sample of this patient from day 28 on-dasatinib to isolate the CD34+/CD14–, resp. CD34(+)/CD14– and the CD34–/CD14+ cohorts for further molecular characterization: Intriguingly, the leukemia-cell specific *KIT* D816V mutation was detected in the CD34 positive as well as CD34 negative cohorts, strongly arguing for a release of the differentiation blockage upon exposure of leukemic blasts to dasatinib *in vivo* (Figure 2F).

Together, these findings strongly argue for *in vivo* antileukemic efficacy of dasatinib monotherapy in this mutant-*KIT*, CBFβ-MYH11 positive patient.

The molecular mechanisms remain to be elucidated: Several reports have suggested a crucial role of CCAAT/enhancer-binding-proteins (C/EBP) in hematopoiesis and deregulation is a common occurrence in leukemogenesis (reviewed e.g. by Ramji and Foci [8] and Publican, Tenen and Behre [9]). C/EBP are subject to alternative translation modulating expression of inhibitory and activating isoforms. It has been shown that wildtype and mutant-FLT3 isoforms activate alternative translation of C/EBPbeta via the PI3K/motor/RSK pathways [10]. While speculative, as (mutant-) KIT is known to signal via the PI3K/AKT cascade as well [4], it is likely that inhibition of the gain-of-function *KIT* D816V isoform (via dasatinib) may alter C/EB-proteins to affect proliferation and differentiation. However, addressing this hypothesis is complex and needs to be answered in future mechanistic studies in detail.

Several problems occurred: Clinical deterioration was noted on day 29, with dyspnea and fever – together with reoccurrence of leukocytosis, suspicious for leukemia relapse and pulmonary infection in neutropenia. However, cytomorphology revealed prevalence of granulocytic and monocytic cells, not blasts. HU was readministered starting on day 29 (500 mg/d, escalated BID on day 31) leading to quick decline in leukocytosis the following days. Empiric antibiotic treatment for suspected pulmonary infection did not result in clinical improvement.

**Figure 3:**
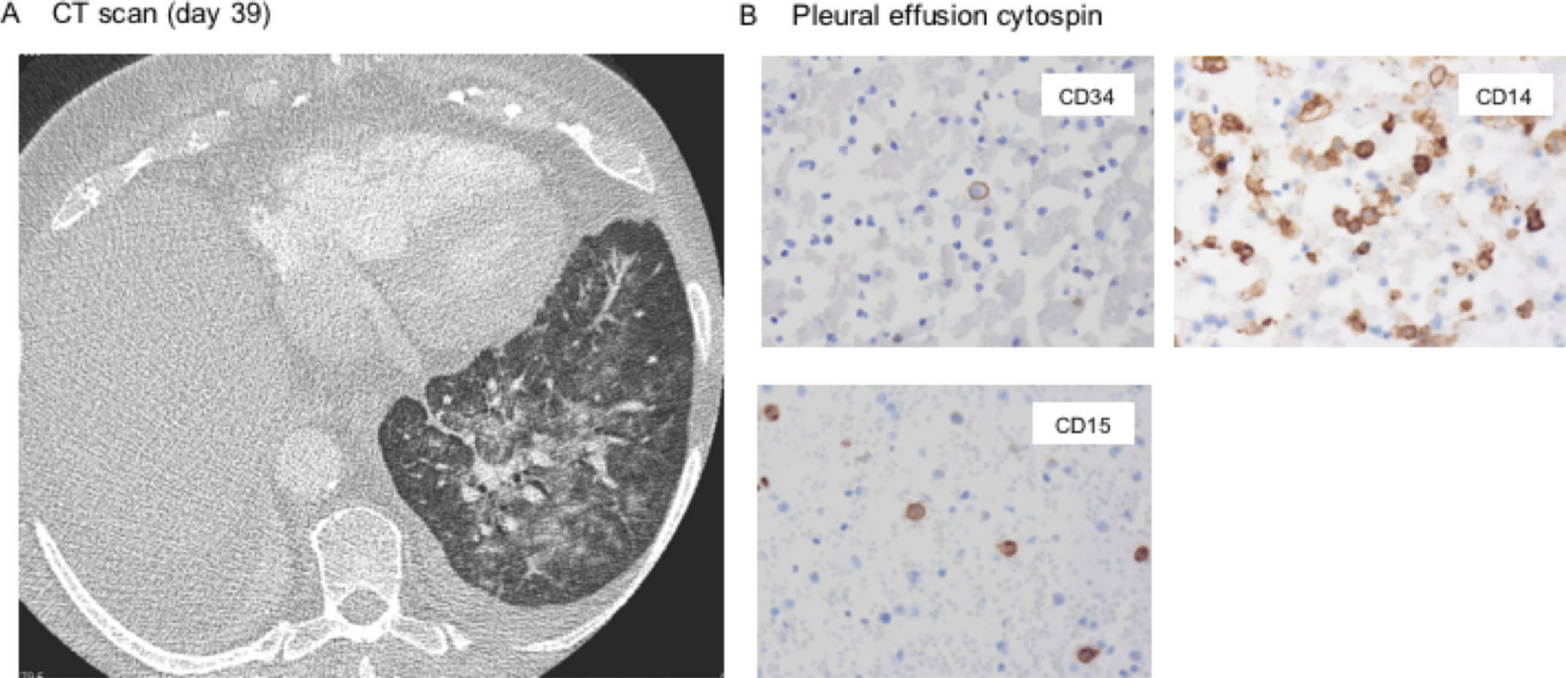
Pulmonary differentiation syndrome triggered by dasatinib (**A**) Chest CT scan arguing for dasatinib-mediated pulmonary differentiation syndrome showing typical infiltrates, signs of noncardiogenic pulmonary edema and pleural effusion. (**B**) Cytospin of a pleural effusion aspirate shows predominance of maturing CD34-negative cells (histology block preps ×400).

Due to progressing clinical deterioration with fever, hypoxia and edema a chest CT scan was performed demonstrating pulmonary infiltrates and pleural effusion consistent with pulmonary differentiation syndrome (Figure 3A).

Whereas the radiographic findings, also resembling engraftment syndrome after stem cell transplantation due to cytokine release during granulocytic expansion and invasion, do not rule out pulmonary hemorrhage in thrombopenia or dasatinib-associated effusion – treatment with dexamethasone pulse therapy quickly improved the symptoms, strengthening the differential diagnosis of dasatinib-induced differentiation syndrome. This observation is further underlined by histomorphologic findings of the pleural effusion aspirate, revealing predominance of CD14+/CD34– differentiated, partly phagocytic (i.e. functionally active) cells (Figure 3B).

In this context it is noteworthy, that pulmonary differentiation syndrome has been described in *CBFβ-MYH11/ABL1* AML – and was reported earlier in two patients with myelomonocytic leukemia (FAB M4eo) with high white blood cell counts at presentation and treated with cytarabine-based induction chemotherapy escalated with all-trans retinoic acid (ATRA) [11].

In our patient, dasatinib was not well tolerated and clinical course was further complicated by upper gastrointestinal bleeding due to thrombopenia (day 36).

Treatment was paused and readministered after recompensation in a reduced dose of 100 mg/day. However, due to persistent inacceptable tolerability of dasatinib in this frail patient (gastrointestinal discomfort associated with drug intake), therapy was permanently stopped in favor of a BSC concept including cytoreduction (HU).

In this context, targeting mutant-KIT feasible but novel inhibitors with improved pharmacodynamics and –kinetics are warranted. It also should be considered that KIT inhibitors may also target wild type KIT, potentially increasing the risk of myelosuppression as discussed earlier by Galanis and Levis [12]. It is notable that we have recently demonstrated that crenolanib, a preferential FLT3 inhibitor compared to KIT inhibition–with less myelosuppressive capacity [13, 14], is a potent mutant-KIT inhibitor-including KIT D816 isoforms [7] providing an attractive agent to be further investigated in mutant-*KIT* malignancies. Also of importance, proof-of-principle and early clinical data of a novel highly-specific *KIT* D816V-inhibitor (BLU-285) were just reported in systemic mastocytosis [15, 16] – offering high translatory potential to mutant-*KIT* acute leukemia.

To summarize, we provide proof-of-principle that KIT inhibition in CBFβ-MYH11, mutant-*KIT* positive CBF AML has clinical antileukemic efficacy. Interestingly, besides cytoreductive properties, the potentially clinically more challenging mode-of-action may be release of the differentiation blockage of leukemia blasts – which needs special attention to prevent misdiagnosis. Our observations are thereby in line with a previous case report of a patient with RUNX1/RUNX1T1 positive, KIT wildtype AML responding to dasatinib [17].

To conclude, targeting KIT in CBF AML has clinical efficacy – but needs close clinical monitoring and supportive care.

## SUPPLEMENTARY MATERIALS AND FIGURES



## Abbreviations

AML: acute myeloid leukemia
BID: two times a day
BSC: best supportive care
CBF: core binding factor
HU: hydroxy urea
PIA: plasma inhibitory assay
TID: three times a day
TKI: tyrosine kinase inhibitor.

## References

[1] Papaemmanuil E, Gerstung M, Bullinger L, Gaidzik VI, Paschka P, Roberts ND, Potter NE, Heuser M, Thol F, Bolli N, Gundem G, Van Loo P, Martincorena I (2016). Genomic classification and prognosis in acute myeloid leukemia. N Engl J Med.

[2] Paschka P, Marcucci G, Ruppert AS, Mrozek K, Chen H, Kittles RA, Vukosavljevic T, Perrotti D, Vardiman JW, Carroll AJ, Kolitz JE, Larson RA, Bloomfield CD (2006). Adverse prognostic significance of kit mutations in adult acute myeloid leukemia with inv(16) and t(8;21): A cancerand leukemia group b study. J Clin Oncol.

[3] Wang YY, Zhao LJ, Wu CF, Liu P, Shi L, Liang Y, Xiong SM, Mi JQ, Chen Z, Ren R, Chen SJ (2011). C-kit mutation cooperates with full-length aml1-eto to induce acute myeloid leukemia in mice. Proc Natl Acad Sci USA.

[4] Schittenhelm MM, Shiraga S, Schroeder A, Corbin AS, Griffith D, Lee FY, Bokemeyer C, Deininger MW, Druker BJ, Heinrich MC (2006). Dasatinib (bms-354825), a dual src/abl kinase inhibitor, inhibits the kinase activity of wild-type, juxta membrane, and activation loop mutant kit isoforms associated with human malignancies. Cancer Res.

[5] Shah NP, Kantarjian HM, Kim DW, Rea D, Dorlhiac-Llacer PE, Milone JH, Vela-Ojeda J, Silver RT, Khoury HJ, Charbonnier A, Khoroshko N, Paquette RL, Deininger M (2008). Intermittent target inhibition with dasatinib 100 mg once daily preserves efficacy and improves tolerability in imatinib-resistant and -intolerant chronic-phase chronic myeloid leukemia. J Clin Oncol.

[6] Kampa-Schittenhelm KM, Heinrich MC, Akmut F, Dohner H, Dohner K, Schittenhelm MM (2013). Quizartinib (ac220) is a potent second generation class iii tyrosine kinase inhibitor that displays a distinct inhibition profile against mutant-flt3, -pdgfra and -kit isoforms. Mol Cancer.

[7] Kampa-Schittenhelm KM, Frey J, Haeusser LA, Illing B, Pavlovsky AA, Blumenstock G, Schittenhelm MM (2017). Crenolanib is a type I tyrosine kinase inhibitor that inhibits mutant kit d816 isoforms prevalent in systemic mastocytosis and core binding factor leukemia. Oncotarget.

[8] Ramji DP, Foka P (2002). Ccaat/enhancer-bindingproteins: Structure, function and regulation. Biochem J.

[9] Pulikkan JA, Tenen DG, Behre G (2017). C/ebpalphaderegulation as a paradigm for leukemogenesis. Leukemia.

[10] Haas SC, Huber R, Gutsch R, Kandemir JD, Cappello C, Krauter J, Duyster J, Ganser A, Brand K (2009). Itd- and fl-induced flt3 signal transduction leads to increased c/ebpbeta-lip expression and lip/lap ratio by different signalling modules. Br J Haematol.

[11] Lester WA, Hull DR, Fegan CD, Morris TC (2000). Respiratory failure during induction chemotherapy for acute myelomonocytic leukaemia (fab m4eo) with ara-c and all-trans retinoic acid. Br J Haematol.

[12] Galanis A, Levis M (2015). Inhibition of c-kit by tyrosine kinase inhibitors. Haematologica.

[13] Galanis A, Ma H, Rajkhowa T, Ramachandran A, Small D, Cortes J, Levis M (2014). Crenolanib is a potent inhibitor of flt3 with activity against resistance-conferring point mutants. Blood.

[14] Smith CC, Lasater EA, Lin KC, Wang Q, McCreery MQ, Stewart WK, Damon LE, Perl AE, Jeschke GR, Sugita M, Carroll M, Kogan SC, Kuriyan J (2014). Crenolanib is a selective type i pan-flt3 inhibitor. Proc Natl Acad Sci U S A.

[15] Rapid responses to avapritinib (blu-285) in mastocytosis (2017). Cancer Discov.

[16] Evans EK, Gardino AK, Kim JL, Hodous BL, Shutes A, Davis A, Zhu XJ, Schmidt-Kittler O, Wilson D, Wilson K, DiPietro L, Zhang Y, Brooijmans N (2017). A precision therapy against cancers driven by kit/pdg framutations. Sci Transl Med.

[17] Chevalier N, Solari ML, Becker H, Pantic M, Gartner F, Maul-Pavicic A, Hubner J, Wasch R, Schmitt-Graff A, Lubbert M (2010). Robust *in vivo* differentiation of t(8;21)-positive acute myeloid leukemia blasts to neutrophilic granulocytes induced by treatment with dasatinib. Leukemia.

