# Supplementary material for: Dasatinib overrides the differentiation blockage in a patient with mutant-KIT D816V positive CBFβ-MYH11 leukemia
Source: Oncotarget. 2018 Jan 31;9(14):11876–82. doi: 10.18632/oncotarget.24376 (PMC5837740; doi:10.18632/oncotarget.24376)
Supplement: Supplementary file 1 [file oncotarget-09-11876-s001.pdf]

# Dasatinib overrides the differentiation blockage in a patient with mutant-*KIT* D816V positive CBFβ-MYH11 leukemia

## SUPPLEMENTARY MATERIALS

A

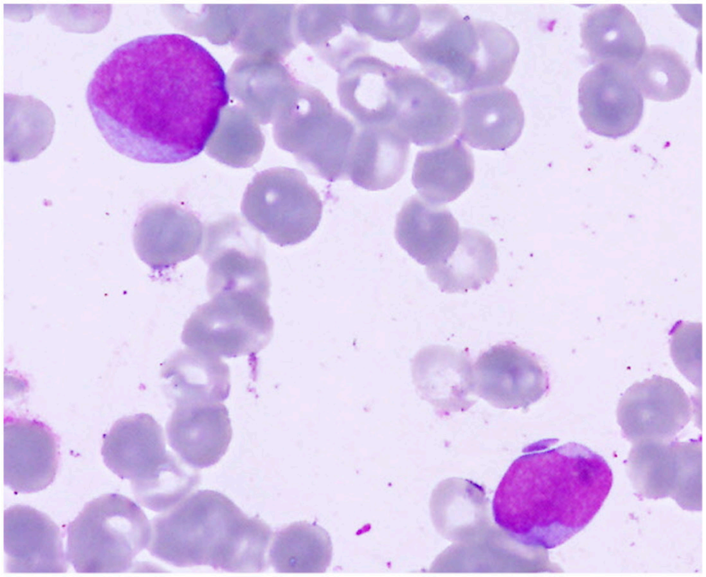

B

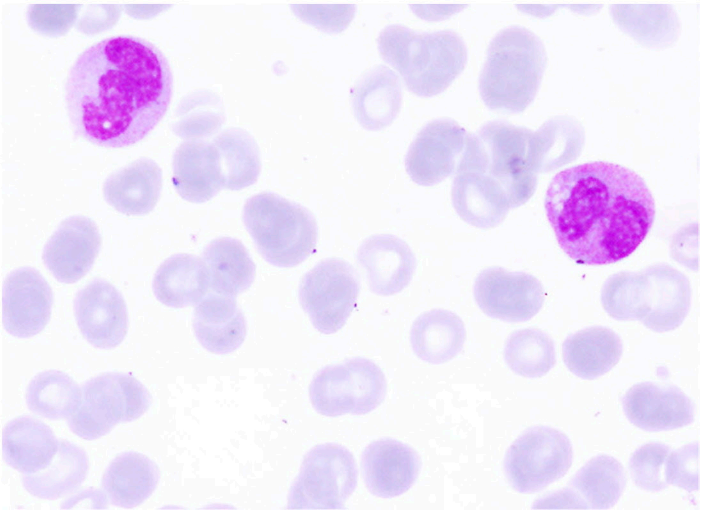

**Supplementary Figure 1: Zoom-in figures of release of the maturation blockage in leukemia cells in response to dasatinib (compare Figure 2A/2B).** (**A**, **B**) Cytology (confocal Zeiss® AXIO Imager.A1 microscope): A May-Gruenwald-Giemsa stain shows signs of dysplastic maturation of granulo and monocytoid-like cells (A, untreated patient blood smear prior to start of treatment with dasatinib; B, 28-day on-dasatinib patient sample).
